# A Comparative Study Between the Effectiveness of 980 nm Photobiomodulation Delivered by Hand-Piece With Gaussian vs. Flat-Top Profiles on Osteoblasts Maturation

**DOI:** 10.3389/fendo.2019.00092

**Published:** 2019-02-20

**Authors:** Reem Hanna, Dimitrios Agas, Stefano Benedicenti, Sara Ferrando, Fulvio Laus, Vincenzo Cuteri, Giovanna Lacava, Maria Giovanna Sabbieti, Andrea Amaroli

**Affiliations:** ^1^Department of Surgical and Diagnostic Sciences, Laser Therapy Centre, University of Genoa, Genoa, Italy; ^2^Department of Oral Surgery, Dental Institute, King's College Hospital NHS Foundation Trust, London, United Kingdom; ^3^School of Biosciences and Veterinary Medicine, University of Camerino, Camerino, Italy; ^4^Laboratory of New Model Organism (NeMo LAB), Department of Earth, Environmental and Life Sciences, University of Genoa, Genoa, Italy

**Keywords:** photobiomodulation, bone regeneration, osteoblast, Low Level Laser Therapy (LLLT), light therapy, cell proliferation

## Abstract

Photobiomodulation (PBM) is a clinically accepted tool in regenerative medicine and dentistry to improve tissue healing and repair and to restore the functional disability. The current *in vitro* study aimed to investigate the photobiomodulatory effects of 980 nm wavelength (the real energy at the target: ~0.9 W, ~0.9 W/cm^2^, 60 s, ~55 J/cm^2^ and a single energy ~55 J in CW) on MC3T3-E1 pre-osteoblast, delivered with flattop profile in comparison to the standard profile. The laser groupings and their associated energies were: Group 1 - once per week (total energy 110 J); Group 2 - three times per week (alternate day) (total energy 330 J); Group 3 - five times per week (total energy 550 J). The metabolic activity and the osteoblasts maturation were analyzed by alkaline phosphatase assay, alizarin red S histological staining, immunoblot and/or double immunolabeling analysis for Bcl2, Bax, Runx-2, Osx, Dlx5, osteocalcin, and collagen Type 1. Our data, for the first time, prove that laser irradiation of 980 nm wavelength with flat-top beam profile delivery system, compared to standard-Gaussian profile, has improved photobiomodulatory efficacy on pre-osteoblastic cells differentiation. Mechanistically, the irradiation enhances the pre-osteoblast differentiation through activation of Wnt signaling and activation of Smads 2/3-βcatenin pathway.

## Introduction

Bone tissue regeneration remains a challenge in the field of oral and maxillofacial surgery reconstruction. Bone defects are the major causes of functional and aesthetical disability, which have a great impact on patients' life quality. One of the main goals in treating a bony defect is to restore the normal morphology and the function of the affected region (1, 2). Bone autografts have been considered to be the gold standard for bone grafting due to their unique characteristics in containing osteogenic cells and bone matrix proteins, which support the bone growth (2, 3). However, they are associated with post-operative complications in terms of donor morbidity, which has been estimated approximately 30,000 patients per year worldwide (1). To date, numerous novel adjunctive methods have been reported to facilitate and accelerate the healing process and shorten the treatment duration, in order to reduce patient's morbidity and to enhance patient's experience (1–3). Photobiomodulation (PBM) can be considered as one of them. Indeed, PBM is a clinically accepted tool in regenerative medicine and dentistry to improve tissue healing and repair and restore the functional disability (3–5). Evidence based-medicine has shown that PBM carries a minimal to none adverse effects on patients' health status in comparison to other tissue regenerative modalities (3, 5, 6).

Photobiomodulation concept operates on cell manipulation through, which the photonic energy transferred by means of light sources (either the coherent light of lasers or the non-coherent energy of LEDs) to trigger the redox biological-chemical reactions, when the oxidation state of atoms is changed (5–7). In this context, the cell metabolism can be modulated, leading to secondary effects, which modifies the cellular behavior (8). Despite of *in vitro* and *in vivo* studies (animal models and randomized controlled clinical trials) with positive PBM outcomes, it still remains a controversial subject as a consequence of the conflicting effects produced by various operating parameters such as wavelength, power output, exposure time (9) and the beam profile (10) as well as the cellular hormetic (non-linear) dose-response (10, 11). For instance, in this perspective, most of the *in vitro* studies applied PBM, utilized wavelengths in a range 600–700 nm (12). However, through *in vivo* studies, the light-energy in this range of wavelengths can quickly disperse and does not penetrate into the deeper target tissue layers, in order to induce therapeutic effects. Conversely, wavelengths ranging from 800 up to 1100 nm have a longer optical penetration depth, which can target deeper tissues (13, 14). The precise mechanism of PBM has not been completely explained and fully understood. Basically, the PBM events can be broadly divided into primary and secondary events (6). The primary laser interaction (sub-microsecond range) at 600–810 nm has been described to act on the cellular chromophores, prevalently located in the mitochondria (6, 15, 16). The primary indirect event (seconds-minutes duration) involves the mitochondria oxygen consumption, reactive oxygen species (ROS) production and the ATP synthesis (15–18). While the mechanistic sequence of the secondary events (hour-days duration) is to date contradictory and poorly understood due to a lack of standardization of the experimental studies and the focus was predominately on the macroscopic phenomenological effects (6, 19). This has been documented in the systematic review by Deana et al. (12), which reported that the osteoblasts-like cells were responsive to the effects of the PBM. However, most of the laser parameters utilized in the studies of this literature review have varied by different authors. This has led to little or none influence on proliferation of the cells, whilst the high irradiance has demonstrated deleterious effects on the proliferation of the cells (12). Equally, Amid and co-worker (19) have shown that the PBM at a low energy density exerted bio-stimulatory effects on the proliferation and the differentiation of the bone cells but no evidence to suggest its precise mechanism of action. This discrepancy in the effects of PBM on the osteoblast-like cells proliferation has highlighted the bi-phasic effect of PBM. Amaroli et al. (10, 15, 18) have shown that a reconsideration for a higher-fluence with a higher-energy should be addressed, due to the advances in the field of the bio-photonic technologies in utilizing the flat-top (FT) profile delivery system instead of the Gaussian profile (Standard (ST) hand-piece). As a matter of fact, studies have shown that unicellular model irradiation, *Paramecium primaurelia*, with 808 nm wavelength using a FT profile delivery system (AB2799 FT) at a power setting of 1 W over 1 cm^2^ for 60 s exposure time (power density 1 W/cm^2^, energy density 60 J/cm^2^) in continuous wave (CW) mode, has positive effects on the cell and tissue metabolism (15, 20). This occurred by stimulating the activities of the mitochondria (oxygen consumption; ATP production) (10, 18), calcium fluxes (21, 22), nitric oxide production (22) and the cell proliferation (23) in *Paramecium primaurelia*. Moreover, this has shown positive effects on human endothelial cell (24) and murine bone marrow stromal cells differentiation in enhancing osteogenesis (25). That higher laser energy, against expectation, did not uncouple the mammal mitochondria respiratory chain (18) and did not generate any genotoxic effects (26) but on the contrary it promoted the wound healing via the inflammatory processes decrement in the animal models (27).

Phobiomodulation at an appropriate fluence, wavelength, power output and frequency of irradiation can generate biostimulative effects on the pre-osteoblast cells, thereby causing a shift in the cell to an activated stage in the cell cycle (12). However, there is a gap in literature, concerning what happens inside an individual cell type, osteoblast, when it is exposed to a particular light source (6, 19). It is only through a better scientific understanding research of the underlying mechanisms, as well as optimization of irradiation parameters that this therapy will be fully accepted by the medical profession.

On the basis described above, the current *in vitro* study, aimed to investigate and evaluate for the first time the photobiomodulatory effects of 980 nm wavelength on MC3T3-E1 pre-osteoblast cells at a higher fluence delivered by hand-piece with FT profile in comparison to the ST profile.

The objectives of this study were as follow: (1) The primary objective was to determine the optimal laser parameters of the 980 nm wavelength that exerts bio-stimulatory to accelerate and enhance the bone regenerative process; (2) The secondary objective was to evaluate the intra-cellular pathways of the photon-cell interaction across the metabolic, proliferative and differentiation changes, which ultimately lead to the bone healing and repair. (3) Lastly, a comparison between the effectiveness of irradiation with FT profile vs. ST profile was analyzed.

## Materials and Methods

### Cell Culture

The MC3T3-E1 cell line utilized in the current study (mouse calvarian pre-osteoblasts), (ATCC, LGC Standards S.r.L Milano, Italy) were grown in Minimum Essential Medium Eagle (αMEM) (Life Technologies Milano, Italy) supplemented with 10% heat inactivated fetal calf serum (HI-FCS) (Life Technologies Milano, Italy) penicillin (100 U/ml), and streptomycin (50 μg/ml) (Life Technologies Milano, Italy).

### The Irradiation Tools and Laser Power Out Measurements

In current study, 980 nm diode laser (Doctor Smile–LAMBDA Spa–Vicenza, Italy) with two delivery systems was utilized; the standard hand-piece (Gaussian profile) (ST) and flat-top (FT) profile hand-piece utilized to irradiate the MC3T3-E1 pre-osteoblast cells. All the equipment purchased at the Doctor Smile–LAMBDA Spa–Vicenza, Italy.

### The Laser Protocol and Experiments to Obtain Approximately Similar Power Output for Both Delivery Systems (FT and ST)

The design of the laser irradiation is shown by Table 1A.

**Table 1A:** Design of the laser irradiation for the experimental set-up with power-meter.

**Air (^**a**^)**	**Petri-dish (^**b**^)**	**αMEM + Petri dish (^**c**^)**
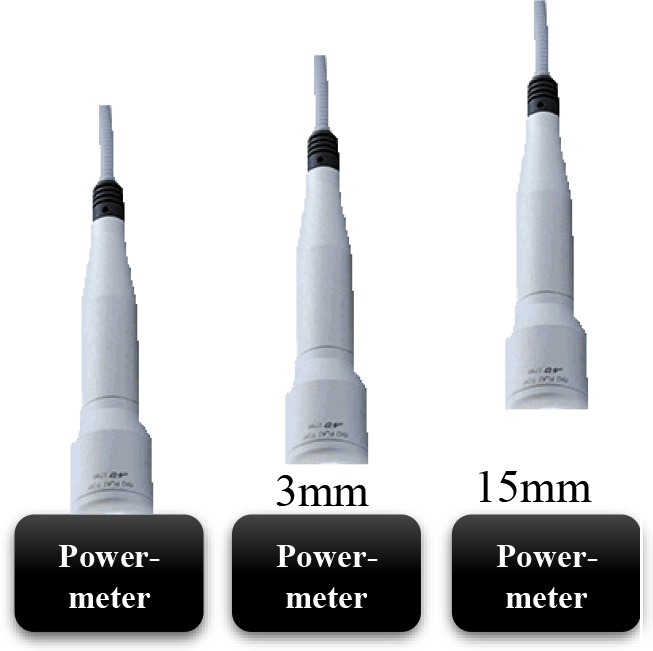	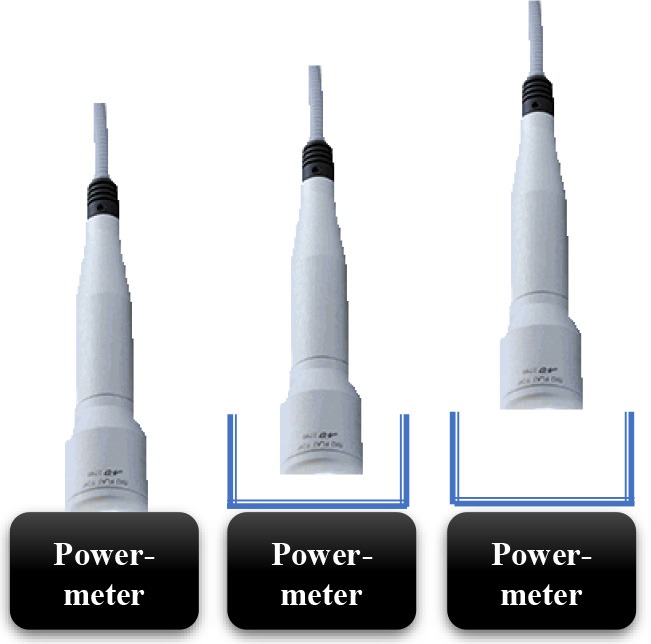	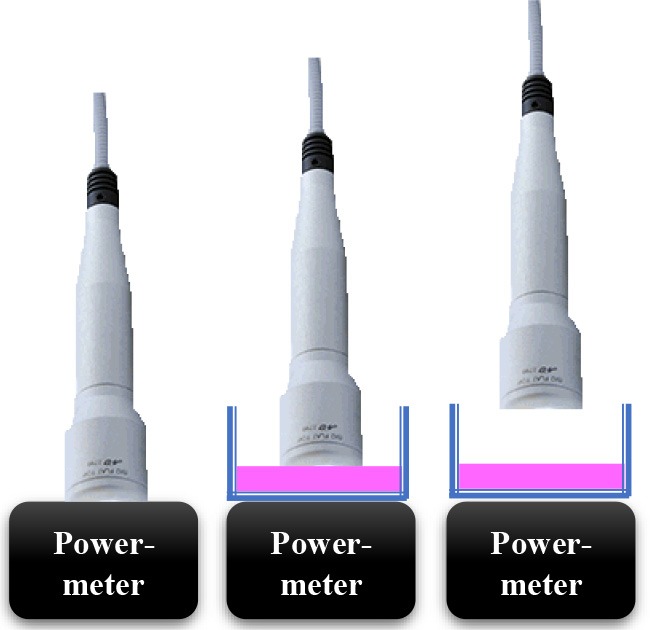

Our initial laser parameters were based on our previous work (22). The power out was set at 1 W power output in CW at exposure time of 60 s. The power density was 1 W/cm^2^ and the fluence was 60 J/cm^2^. In order to obtain our accurate laser parameters, taking in consideration the light attenuation through the medium and materials used in the experiments, the PM160T-HP power-meter (ThorLabs, Germany) was utilized in this study. It assisted to measure the power output in a continuous wave when the laser light passes through various materials and culture medium. Therefore, the hand-pieces (the delivery system) of the FT and the ST profiles were setup at a distance of 3 and 15 mm away from the center of the power meter where the sensor is located. Based on the above descriptions (Table 1A), the cellular experiments were setup at 3 mm distance when an empty petri dish utilized without its cover and when the petri dish (without the cover) filled with 3 mm thickness of αMEM (see scheme in the Table 1A). Based on the above outcomes, the laser device was setup with the precise laser parameters for this cellular experiment, which were measured with power meter are as follow: 1 W, 1 W/cm^2^, 60 s in CW, 60 J/cm^2^ for the FT hand-piece irradiations and 1.1 W, 1.1 W/cm^2^, 60 s in CW, 66 J/cm^2^ for the ST hand-piece irradiations. This clearly demonstrated that the cells were irradiated with no statistical differences at a power output of 0.93 ± 0.02 W and 0.90 ± 0.02 W (*P* < 0.05) respectively (Table 2).

### The Culture Irradiation Protocol

The cultures irradiated according to the laser parameters described in Tables 1B, 2 and with the setup of our previous study (25). The frequencies of the irradiation were based on once, three times (alternate day) and five times (every day) per week for duration of two consecutive weeks. The exposure time was 60 s. This protocol applied to the ST and FT delivery systems of 980 nm diode laser therapy.

**Table 1B:** Measure of the power laser energy (Watt, W) in the experimental set-up.

	**Wavelength (nm)**	**Set-up's energy (laser device) (W)**	**Output energy (contact) (W)**	**Coming down energy (air, 3 mm)**	**Coming down energy (air, 1.5 cm)**
**AIR (^a^)**					
Standard hand-piece	980	1	0.98 ± 0.03	0.87 ± 0.01 W^*^^/^^**^	0.78 ± 0.02 W^*^^/^^**^
Flat-top hand-piece	980	1	1.01 ± 0.02	1.00 ± 0.01 W^**^	1.02 ± 0.03 W^**^
	**Wavelength (nm)**	**Set-up's energy** **(laser device) (W)**	**Coming down energy** **(air, 3 mm)**	**Coming down energy** **(air, petri, 3 mm)**	
**PETRI DISH (^b^)**					
Standard hand-piece	980	1	0.87 ± 0.01 W^*^^/^^**^	0.79 ± 0.02 W^+^^/^^*^^/^^**^	
Flat-top hand-piece	980	1	1.00 ± 0.01 W^**^	0.91 ± 0.02 W^+^^/^^*^^/^^**^	
	**Wavelength (nm)**	**Set-up's energy** **(laser device) (W)**	**Coming down energy** **(air, 3 mm)**	**Coming down energy** **(air, αMEM, petri, 3 mm)**	
**αMEM + PETRI DISH (^c^)**					
Standard hand-piece	980	1	0.87 ± 0.01 W^*^^/^^**^	0.72 ± 0.02 W^+^^/^^*^^/^^**^	
Flat-top hand-piece	980	1	1.00 ± 0.01 W^**^	0.85 ± 0.02 W^+^^/^^*^^/^^**^	
	**Wavelength (nm)**	**Set-up's energy** **(laser device) (W)**	**Coming down energy** **(air, 3 mm)**	**Coming down energy** **(air, αMEM, 3 mm)**	
**ENERGY THAT ARRIVES TO THE CELL MONOLAYER**					
Standard hand-piece	980	1	0.87 ± 0.01 W^*^^/^^**^	0.80 ± 0.02 W^+^^/^^*^^/^^**^	
Flat-top hand-piece	980	1	1.00 ± 0.01 W^**^	0.93 ± 0.02 W^+^^/^^*^^/^^**^	

*Variation of energy depending by the target distance and the features of the material crossed are reported. The laser device set-up was: 1 W, irradiation time of 60 s, in continuous wave (CW) mode*.

* *Difference statistically significative respect to the output energy of laser device: p < 0.001*.

** *Difference statistically significative between Standard hand-piece vs. Flat-top hand-piece: p < 0.001*.

+ *Difference statistically significative respect to the Coming down energy (Air, 3 mm): p < 0.001*.

**Table 2:** Laser irradiation for the experimental set-up.

	**Wavelength (nm)**	**Set-up's energy** **(laser device) (W)**	**Coming down** **energy (3 mm)**	**Time**	**Fluence**	**Mode**
Standard hand-piece	980	1.1	0.90 ± 0.02 W^*^	60 s	54.2 ± 1.0 J/cm^2^	CW
Flat-top hand-piece	980	1	0.93 ± 0.02 W^*^	60 s	55.8 ± 1.2 J/cm^2^	CW
	**Power density (W/cm**^**2**^**)**	**Time for irradiation (s)**	**No. of irradiation (for 2 weeks)**		**Total dose (J)**	
Standard hand-piece	1.1	60	Once per week		110	
Flat-top hand-piece	1	60	Once per week		110	
Standard hand-piece	1.1	60	Three times per week		330	
Flat-top hand-piece	1	60	Three times per week		330	
Standard hand-piece	1.1	60	Five times per week		550	
Flat-top hand-piece	1	60	Five times per week		550	

* *Difference statistically significative respect to the set-up's energy of laser device: p < 0.001*.

** *Difference statistically significative between Standard hand-piece vs. Flat-top hand-piece: p < 0.001*.

The samples were subdivided as follow:

FT hand-piece group
Group 1- once per week (total energy 110 J).
Group 2 - three times per week (alternate day) (total energy 330 J).
Group 3 - five times per week (total energy 550 J).
The ST hand-piece group
Group 1- once per week (total energy 110 J).
Group 2 - three times per week (alternate day) (total energy 330 J).
Group 3 - five times per week. (total energy 550 J).

The control cultures were processed in identical conditions except that the laser device was kept off all the time. The total energy was 0 J.

### Assessment of the Metabolic Activity of Viable Cells (MTS)

The metabolic activity of viable cells was determined by the MTS [3-(4,5-dimethylthiazol-2-yl)-5-(3-carboxymethoxyphenyl)-2-(4-sulfophenyl)-2H-tetrazolium]. The MC3T3-E1 cells were plated at a density of 5,000 cells/well on 96-well culture plates until 80% confluent. Then, cells were irradiated according to the above protocol. At the end of the treatments, the cell viability was measured by CellTiter 96(R) AQueous One Solution assay (Promega, Milano, Italy) following the manufacturing instructions.

### BrdU Cell Proliferation Assay

The BrdU assay was performed using “BrdU Cell Proliferation ELISA Kit (colorimetric)” (Abcam, Milano, Italy). The Mc3T3-E1 pre-osteoblasts were plated at the density of 3,000 cell/well on 96-well culture plates. At the end of the treatments, they were labeled with BrdU according to the manufacturer's instructions. Plates were read by spectrophotometry at 450 nm (Tecan Infinite reader; Tecan, Milano, Italy).

### Alkaline Phosphatase (ALP) Assay and Alizarin Red S (ARS) Histological Staining

The MC3T3-E1 pre-osteoblasts were plated at 5 × 10^4^ cells/well in 6-well culture plates and grown until about 80% confluent. The cultures were irradiated as according to the protocol. At the end of each set of irradiations, the ALP staining was performed with a commercial kit (Sigma–Aldrich) following the manufacturer's instructions. The images of the stained cells were scanned using a specific scanner. The quantitative analyses of ALP staining were performed by Image J (NIH free software). With regard of the Alizarin red S histochemistry, the cultured cells (untreated and irradiated cells) were stained to evaluate the mineralized matrix (28). Subsequently, the cells' layers were briefly rinsed with PBS and fixed in 4% PFA diluted in PBS for 25 min at a room temperature. Then, the cultures were stained with 2% Alizarin red S (Sigma-Aldrich) for 30 min at 37°C. The cultures were examined under a light microscope. The quantification of the staining was performed as described by Gregory et al. (29). The stain was desorbed and the collected solutions were distributed on 96-well plates for absorbance reading (triplicate) at 405 nm by spectrophotometry (Tecan Infinite reader; Tecan, Milano, Italy).

### Western Blotting

The MC3T3-E1 pre-osteoblasts that plated at 5 × 10^4^ cells/well in 6-well culture plates were grown and treated as described above. At the end of each set of treatment, the proteins from the irradiated MC3T3-E1 pre-osteoblasts or untreated cells (control) were extracted with cell lysis buffer (Cell Signalling, Milano Italy) and the concentration was determined by the bicinchoninic acid (BCA) protein assay reagent (Pierce, Euroclone Milano, Italy). The Western blotting was performed as previously described (30). Membranes were immunoblotted in blocking buffer with specific antibodies: rabbit anti-runt-related transcription factor 2 (Runx-2) antibody, rabbit polyclonal anti-B-cell lymphoma 2 (Bcl2), rabbit polyclonal anti-Bcl-2-associated X protein (Bax) and rabbit polyclonal anti-osterix (Osx) antibodies (all above were in 1:400 dilution (Santa Cruz Biotechnology, DBA, Milano, Italy); mouse anti-Distal-less homeobox 5 (Dlx5), rabbit monoclonal anti-non-phospho-β-catenin (β-catenin), rabbit monoclonal anti-phospho-Small-mothers against decapentaplegic (Smads) 2/3 (all above were in 1:800 dilution, Cell Signalling, EuroClone, Milano, Italy); rabbit anti-Transforming growth factor β (TGFβ) (1:600, dilution, Abcam, Prodotti Gianni, Milano, Italy). After washing the blots with PBS-T, they incubated with horseradish peroxidase (HRP)-conjugated donkey anti-rabbit immunoglobulin G (IgG) antibody or with HRP-conjugated rabbit anti-mouse IgG (Cell Signaling, Euroclone Milano, Italy) and then diluted at 1:80,000 in a blocking solution for 1 h at room temperature. After further washing of the blots with PBS-T, the immunoreactive bands were visualized using luminol reagents and Hyperfilm-enhanced chemiluminescence film (Euroclone, Milano, Italy) in accordance to the manufacturer's instructions. To normalize the bands, filters were stripped and re-probed with a mouse anti-α-tubulin antibody (Sigma-Aldrich, Milano, Italy). The band density was densitometrically quantified by NIH image.

### Osteocalcin Release in Culture Medium

The MC3T3-E1 cells were plated, grown and treated as described above. At the end of each set of irradiation, the release of osteocalcin in culture medium was quantified by using the mouse osteocalcin ELISA kit (Cusabio, Tema Ricerca srl, Italy) according to the manufacturer instructions and in line with Niu et al. (31).

### Double Immunolabeling for Osteocalcin and Collagen Type I and Fluorescence Analysis

The MC3T3-E1 pre-osteoblasts were plated at 2.5 × 10^4^ cells/well in six-well culture plates containing coverslips, which were previously cleaned and sterilized. The cells were grown and treated in the same manners as described above. Then, the cells were fixed in 4% PFA and permeabilized with 0.3% Triton X-100 for 30 min, incubated with 0.5% bovine serum albumin (BSA) and diluted in PBS for 20 min at room temperature. Then, the cultures were incubated with two antibodies: the rabbit anti-osteoclacin (OC) antibody and the mouse-anti-collagen Type 1 antibody (Col 1a1) (Santa Cruz Biotechnology) antibody in 1:50 dilution in PBS for 2 h (h) at room temperature. After the cells were rinsed, they incubated with a chicken anti-rabbit IgG Alexa Fluor 488 conjugated (*Molecular Probes, Inc. Invitrogen s.r.l., Milano, Italy*) and a goat anti-mouse IgG Alexa Fluor 594 conjugated (*Molecular Probes, Inc*.), which both were diluted at concentration of 1:100 for 1 hr at room temperature. Controls were performed by omitting the appropriate primary antibodies. After the cells were washed, their coverslips were mounted on slides with PBS/glycerol (1:1). Then, the slides were examined utilizing fluorescent microscopy.

Fluorescence analysis was performed by a fluorimeter Tecan Infinite with excitor filter 590 nm and emission 635 nm for Alexa Fluor 594, or 485, and of 535 nm for anti-Alexa Fluor 488. A Tecan Infinite fluorescence reader was utilized to quantify the amount of Alexa Fluor 488-labeled anti-osteocalcin and Alexa Fluor 594-labeled anti-collagen Type I.

### Statistical Analysis

All data were expressed as a mean ± standard error (S.E.). Data were analyzed by using two-way ANOVA followed by Tukey's pairwise comparisons. The letters a, b, c, and d, above a particular column indicate statistically significant difference among. Values of *p* < 0.05 were considered significant. The results are a representative of those acquired by independent experiments repeated at least three times.

## Results

### The Power Out Measurements by Power-Meter

Consistent with the standard error of the instrument, the power-meter measurements in contact mode showed that the power output provided by the hand-pieces (ST vs. FT) were similar (*P* < 0.05) and in accord with the 980 nm laser set-up (~1 W) (Table 1B, output energy, contact). However, with the hand-pieces fixed at 3 and 15 mm distance from the power-meter, only the FT profile hand-piece kept its power output, constant and comparable to the contact mode (*p* < 0.05); 1 ± 0.01 W and 1.02 ± 0.03 W, respectively (Table 1B, coming down energy–air, 3 and 15 mm). While in the ST profile hand-piece, the received power output was progressively decreased, to 0.87 ± 0.01 W (−11%) at 3 mm and 0.78 ± 0.02 W (−20%) at 15 mm. These measures showed (Table 1B, coming down energy–air, petri, 3 mm) a statistically significant decrease (*p* < 0.001) in the power output when both ST and FT hand-pieces irradiated the petri dish base, which were 0.79 ± 0.02 W and 0.91 ± 0.02 W (−9%) respectively. In addition, the energy significantly decreases again because of αMEM and Petri dish (ST = 0.72 ± 0.02; FT = 0.85 ± 0.02) (Table 1B, coming down energy–air, αMEM, petri, 3 mm). Therefore, the effective power output reached the MC3T3-E1 pre-osteoblast cells was 0.80 ± 0.02 W and 0.93 ± 0.02 W (ST vs. FT) (Table 1B, coming down energy–air, αMEM, 3 mm), which showed a decrease by −20 and −7%, respectively in comparison to the 1 W power output set up on the laser device. In order to achieve a similar therapeutic power output (*P* < 0.05) reaching the target the 980 nm laser device was setup at a power output of 1 W irradiation with the FT hand-piece and 1.1 W irradiation with the ST hand-piece. Definitely, the precise power irradiation aimed to reach the pre-osteoblasts was 0.90 ± 0.02 W and 0.93 ± 0.02 W (Table 2, coming down energy−3 mm) at fluences 54.2 J/cm^2^ and 55.8 J/cm^2^ (mean = 55 J/cm^2^) respectively (*P* < 0.05) (Table 2, fluence).

### Effect of Photobiomodulation on Pre-osteoblasts Viability (MTS Assay)

**Figure 1:**
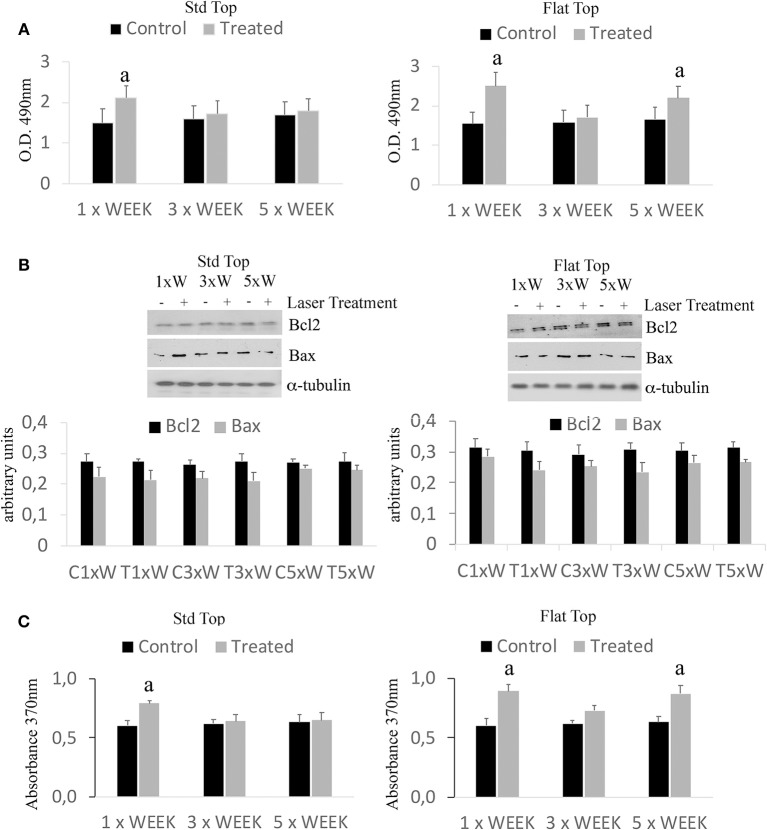
Effect of photobiomodulation on pre-osteoblast viability. **(A)** MTS assay (*n* = 3); **(B)** Western Blotting analysis of B-cell lymphoma 2 (Bcl2) and Bcl-2-associated X protein (Bax) proteins (*n* = 3); **(C)** BrdU cell proliferation assay (*n* = *3*). Bars with different letters show statistically significant difference among the analyzed samples. The values represent means ± S.E; *p* < 0.05, two-way ANOVA followed by Tukey test.

The results have shown only a statistically significant increase (*p* < 0.05) in the cell viability in the group irradiated with the ST hand-piece once a week. However, a statistically significant higher (^*^p < 0.05) in the cell viability showed in the cultures irradiated with the FT hand-piece, once and 5 times per week (Figure 1A). Moreover, similar effects in the ST hand-piece lasered samples in respect to the FT hand-piece irradiated cells (*p* < 0.05) were observed. In order to determine whether the increase in the cell viability with the laser treatment was associated with a modulation of the antiapoptotic protein Bcl-2 or the pro-apoptotic protein Bax, Western blotting analysis was performed. The results showed that the synthesis of these two proteins was not affected by the laser treatment (Figure 1B, *p* < 0.05). Thus, these data evidenced that the increased cell viability, found by MTS assay in laser treated pre-osteoblasts, was likely due to an enhanced cell proliferation as definitely demonstrated by the BrdU assay (Figure 1C, ^*^p < 0.05).

### Effect of Photobiomodulation on Pre-osteoblasts Differentiation

**Figure 2:**
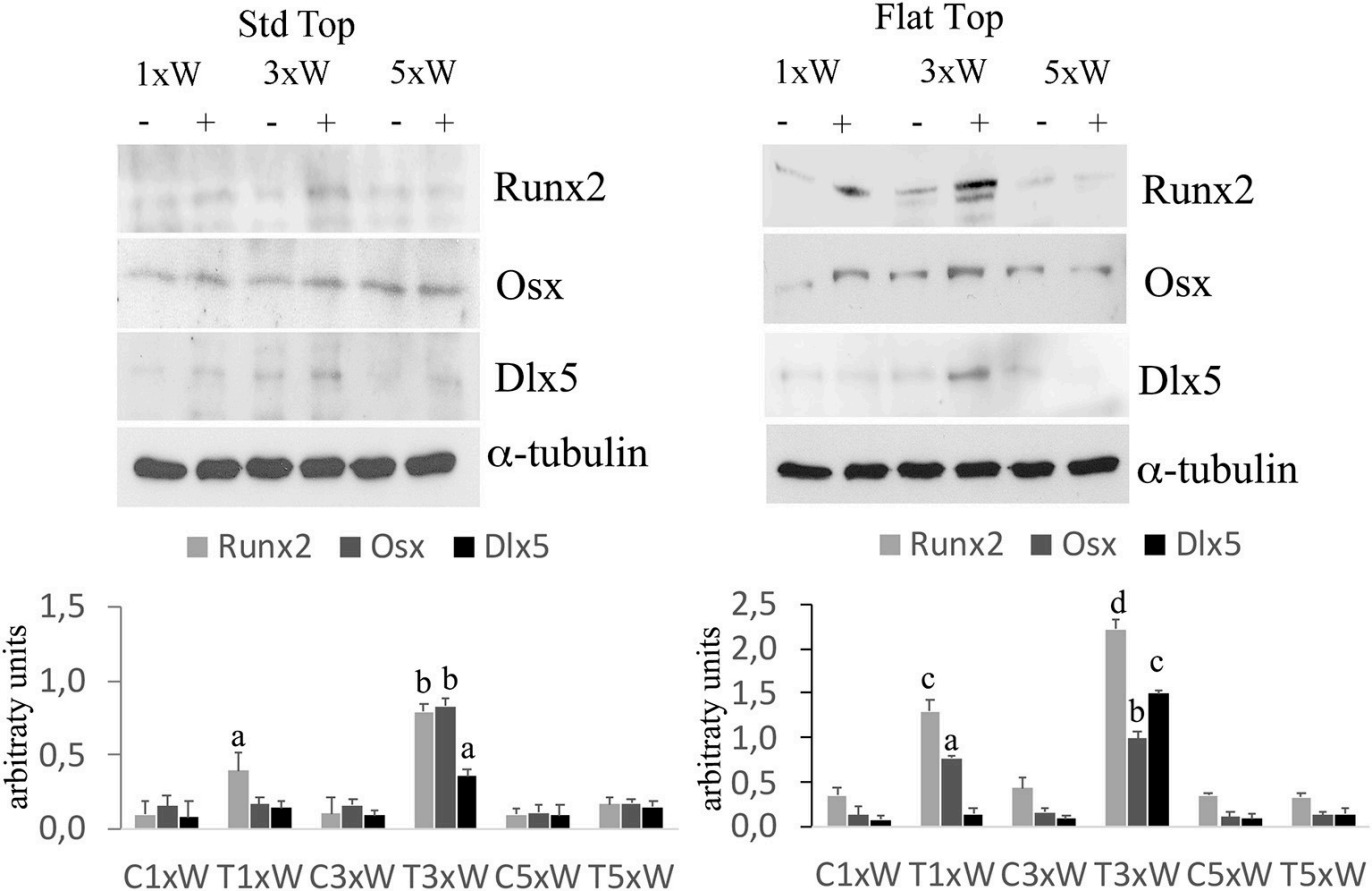
Effect of photobiomodulation on pre-osteoblasts differentiation. Western Blotting analysis of runt-related transcription factor 2 (Runx-2), osterix (Osx) and Distal-less homeobox 5 (Dlx5) proteins (*n* = 3). Bars with different letters show statistically significant difference among the analyzed samples. The values represent means ± S.E; *p* < 0.05, two-way ANOVA followed by Tukey test.

The well-defined signaling network that regulate osteoblasts differentiation comprise transcription factors such as Osx, Dlx5 and Runx2. Our results showed that the irradiated cells with the ST hand-piece once a week for 2 weeks increased the Runx2 protein (*p* < 0.05). However, at the same time point, the increment of Runx2 was higher in the FT hand-piece irradiation (*p* < 0.05) and also the Osx was found incremented in respect to the control group (*p* < 0.05). The employment of laser 3 times a week for 2 weeks with both FT and ST hand-pieces was the only treatment, which increased the Runx2, Osx and Dlx5 proteins expression (*p* < 0.05). Interestingly, the effects on these transcription factors, by the FT hand-piece usage, were markedly and statistically significantly strong in respect to the ST hand-piece (Figure 2).

**Figure 3:**
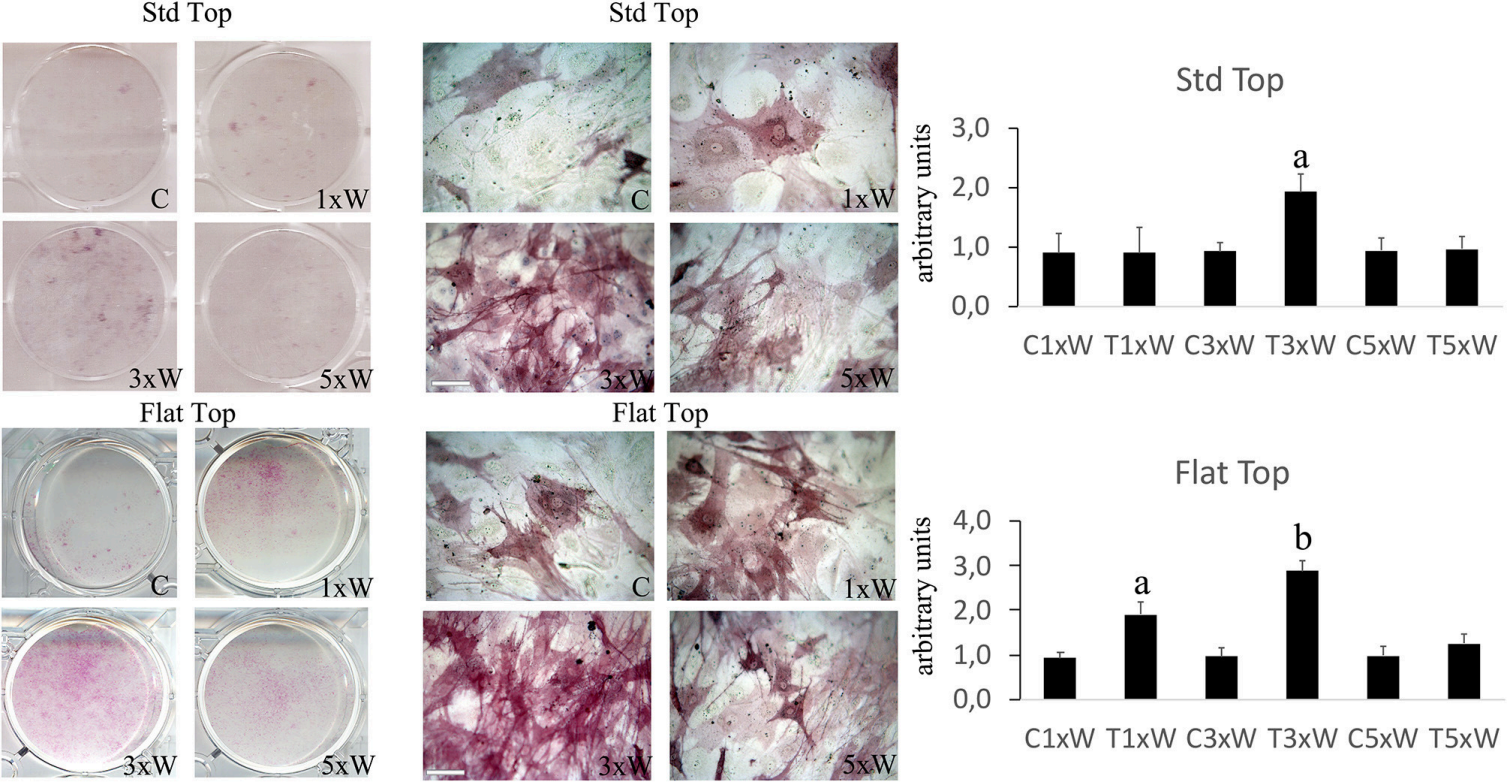
Effect of photobiomodulation on pre-osteoblasts differentiation. The Alkaline phosphatase enzyme activity and the image analysis (*n* = 3). Bars with different letters show the statistically significant differences among the analyzed samples. Values represent means ± S.E; *p* < 0.05, two-way ANOVA followed by Tukey test. Bar 100 μm.

**Figure 4:**
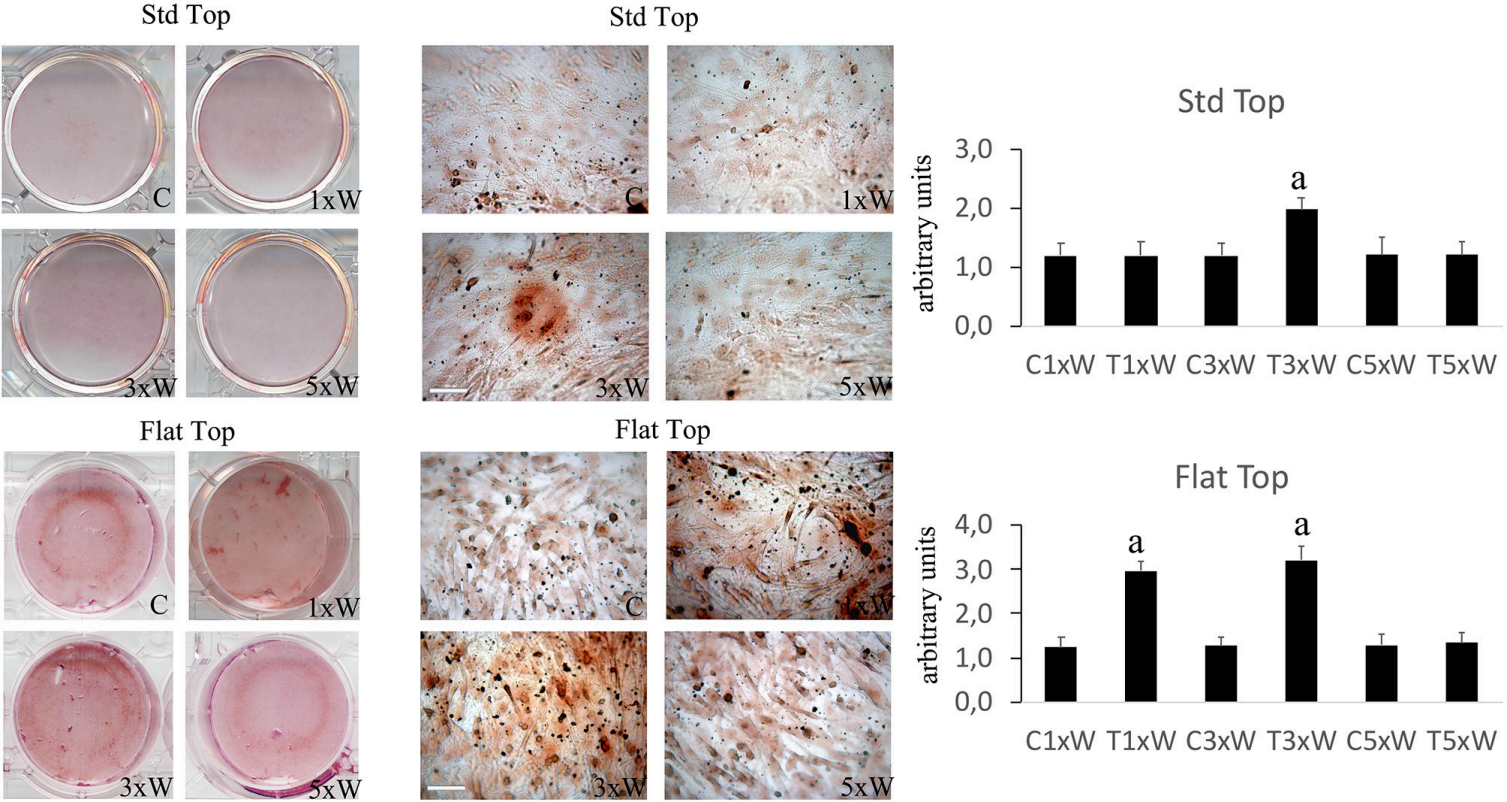
Effect of photobiomodulation on pre-osteoblasts differentiation. Alizarin red staining and image analysis (*n* = 3). Bars with different letters show statistically significant differences among the analyzed samples. The values represent the means ± S.E; *p* < 0.05, two-way ANOVA followed by Tukey test. Bar 100 μm.

The ALP is an enzyme expressed during the early steps of osteoblasts differentiation and it plays important roles in promoting matrix mineralization. A notable increase of both ALP positive colonies (Figure 3) and matrix mineralization (Alizarin red staining) (Figure 4) was observed in the cell cultures irradiated once a week with FT hand-piece. An increment of each distinct staining was observed in 3 times a week irradiation protocol with both FT (Figures 3, 4) and ST hand-pieces (Figures 3, 4). Consistent with the previous results, the use of the FT hand-piece markedly improved the efficacy of the treatment on ALP colonies formation and matrix mineralization (*p* < 0.05).

Mature osteoblasts produce the organic components of the matrix constituted by Col 1a1, OC, osteopontin, osteonectin, and bone sialoproteins.

**Figure 5:**
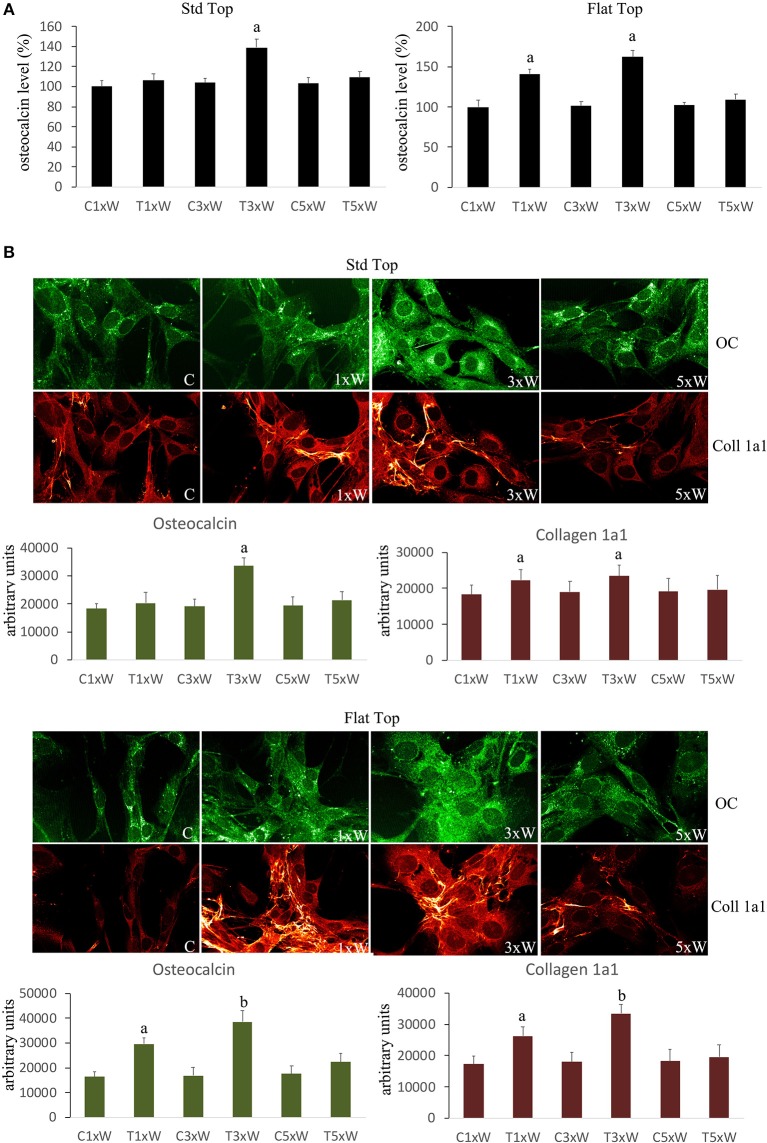
**(A)** Osteocalcin detection in the culture medium (*n* = 3). **(B)** Double immunolabeling for osteocalcin and collagen type 1 (coll 1a1) and the image analysis (*n* = 3). The Fluorescence analysis was quantified by a Tecan Infinite fluorescence reader and values were analyzed by Magellan v4.0 software. Bars with different letters show the statistically significant differences among the samples analyzed. Values represent means ± S.E; *p* < 0.05, two-way ANOVA followed by Tukey test.

The analyses of MC3T3-E1 supernatant as well as immunocytochemical data showed that the OC secretion was statistically up-regulated in cells irradiated with ST hand-piece only in the 3 times a week for 2 weeks protocol. Interestingly, the FT hand-piece irradiation statistically significant incremented OC secretion both at once a week and at 3 times a week irradiation protocol used (Figures 5A,B). The Col 1a1 synthesis was up-regulated by both the ST hand-piece and the FT hand-piece at once a week and 3 times a week treatment. Nevertheless, the FT hand-piece irradiation was found more efficient in inducing this extracellular matrix protein compared with the ST hand-piece (Figure 5B).

### Signalling Pathways Involved in Osteogenic Effects by Photobiomodulation

In osteoblast precursors, the activation of Wnt/β-catenin signaling increases the expression of osteogenic markers. Furthermore, the TGFβ/Smads 2/3 pathway plays an important role in controlling the osteoblasts differentiation and matrix mineralization. Moreover, the BMP2/Smads 1/5/8 was demonstrated as a potent inducer of Runx2, Osx and bone extracellular matrix proteins.

**Figure 6:**
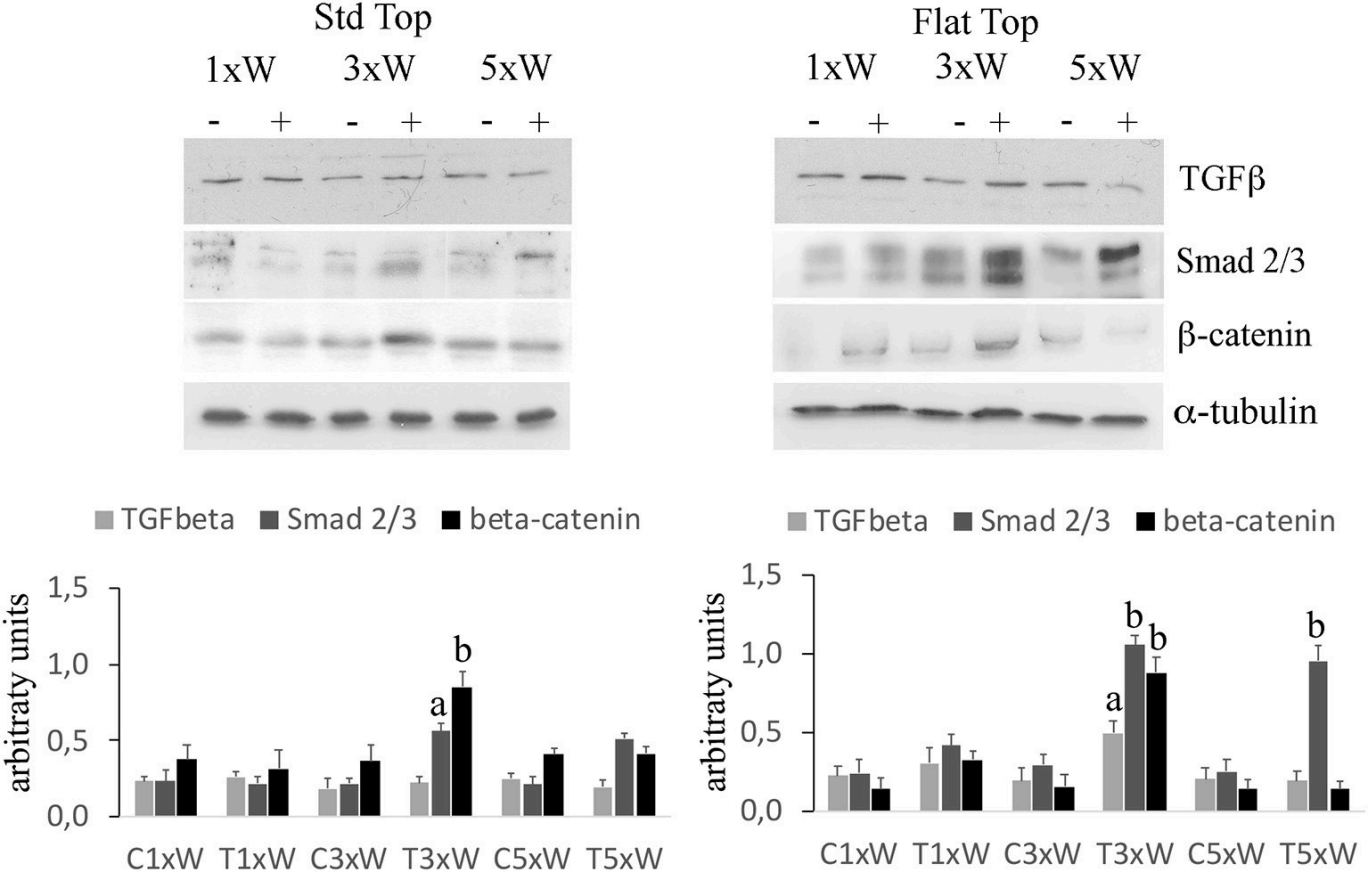
Signaling pathways involved in osteogenic effects by photobiomodulation. Western Blotting analysis of transforming growth factor β (TGFβ) Non-phospho-β-catenin (β-catenin) and phospho-Small-mothers against decapentaplegic (Smads) 2/3 proteins (*n* = 3). Bars with different letters show statistically significant differences among the analyzed samples. The values represent means ± S.E; *p* < 0.05, two-way ANOVA followed by Tukey test.

In our studies, we observed that the laser irradiation of the cells, 3 times a week for 2 weeks, with ST or FT hand-pieces, was able to increase the Smads 2/3 while no effects were noted on the expression of Smads 1/5/8 (data not shown). In addition, an increment of Smads 2/3 was found in cells irradiated five times a week with FT hand-piece. Particularly, while the effect on the Smads 2/3 was similar with both hand-pieces, the irradiation with the FT was also able to increase the β-catenin compared with the ST hand-piece (Figure 6).

## Discussion

Photobiomodulation has been known for almost 50 years but only recently much knowledge has been gained in this area. In fact, literature documentations demonstrated no predictable results about the molecular, the cellular and the tissular mechanisms of action. In addition, the variation of different laser parameters would influence the reliability and the repeatability of the result. This would limit its widespread acceptance (32). In this context, our data, for the first time, showed that if the irradiation was performed with the ST hand-piece, the laser parameter setup on the laser device would not represent the precise optimal power reaching the target site at non-contact distance (contact = 1 W; 3 mm = 0.87 W; 15 mm = 0.78 W). Conversely, the same power output (contact = 1 W; 3 mm = 1 W; 15 mm = 1.01 W) can reach the cellular target site when the reliable homogenous FT hand-piece irradiation utilized. Our *in vitro* data clearly demonstrated that the medium and the materials that were utilized in these experiments, taking in consideration the distance between the laser beam and the center of the power meter sensor, have a great influence on the amount of the photonic energy reaching the target cells. It is important to consider that even if the light travels through few millimeters (mm) thickness of a standard medium, it will have an influence on the PBM effects in the cellular experiments. Consequently, it would reduce the optimal therapeutic power by −20% with the ST hand-piece and by −7% with the FT hand-piece irradiations. Therefore, we investigated the other possible differences between ST and FT beam profiles irradiations, by utilizing two different power settings for our cellular experiments, aiming to irradiate the cells with the same effective and therapeutic power output (power ~0.9 W, power density ~0.9 W/cm^2^, time of irradiation 60 s, fluence ~55 J/cm^2^, and a single energy ~55 J in CW). Our data showed that the 980 nm laser therapy increased the pre-osteoblasts viability. In detail, the comparison of these results (as well as the subsequent) with the literature is hindered by a considerable heterogeneity of the irradiation parameters as well as the methods utilized to evaluate the results and the type of osteoblast-like cell cultures. In line with our data, we observed that the irradiation with 980 nm wavelength at fluences of 7.87, 15.74, 39.37, 78.75 J/cm^2^ in CW was able to increase the MC3T3-E1 cell proliferation. However, the irradiation at a distance of 10 cm from the cells, without a FT hand-piece and a no-exhaustive description of the laser energy measurement, did not allow a more thorough comparison. In addition, Huertas et al. (33) noted that 940 nm wavelength in a pulsed mode at a power setting of 70 mW (0.5–1–1.5 W/cm^2^) showed an increase in the proliferation of osteosarcoma cell lines MG-63, whilst they were decreased at a power density of 2–2.5 W/cm (34). Furthermore, utilization of 940 nm wavelength at a power setting of 300 mW in CW (higher power output) was able to stimulate the cell fission rate of human fetal osteoblast cell line (35). This demonstrates that even the wavelengths ranging between 940 and 980 nm are able to interact and stimulate the pre-osteoblast and the osteoblast cells multiplication, despite of the presence of the non-linear dose response and some of total doses employed in our results did not affect that cellular process.

The process of the *in vitro* pre*-*osteoblasts maturation, mimicking their *in vivo* behavior, passes through three distinct stages of development: proliferation, early differentiation (maturation) and late differentiation (mineralization) (12). A complex signaling network and intrinsic transcription factors, including Osx, Dlx5, and Runx2, regulate the early differentiation of the osteoblasts. The Runx2 and Osx are the most important regulators; indeed, the absence of Runx2 mice would inhibit the osteoblasts differentiation (36, 37). Our results showed that the laser irradiation protocol, with both the FT and the ST hand-pieces, 3 times a week for two weeks (total dose of 330 J) was able to increase the Runx2, Osx and Dlx5. Interestingly, the effects of the laser irradiation with this protocol on these transcription factors were strongly and statistically significantly increased when the FT hand-piece was utilized. Therefore, our 980 nm laser irradiation protocol is able to promote the MC3T3-E1 cell differentiation. However, during mesenchymal stem cells commitment, the Runx2 were expressed in bipotential progenitors, which have the possibility to differentiate into both the chondroblasts and in osteoblasts. Certainly, the Osx-null embryos have a normal cartilage development, but they completely lack of bone formation (38). In our results, the concomitant expression of Osx is defined. Therefore, this would lead to osteoblasts differentiation. Moreover, in consistent with previous results on bone marrow stromal cells (25), our data showed that the irradiation of the cells 3 times a week for 2 weeks protocol (total energy of 330 J) by both the hand-pieces has improved the efficacy of the treatment by increasing the ALP colonies formation. The irradiation of the cells once a week for 2 weeks protocol (total energy of 110 J) was able to induce Runx2, Osx, ALP and matrix mineralization, in addition to its effect on cell differentiation when FT hand-piece was utilized. However, only a punctiform effect on Runx2 is detected when the cells irradiated with ST hand-piece. Therefore, total energy of 110 J when both the hand-pieces utilized, has influenced early differentiation markers. Interestingly, when the process was carried out until the mineralization, the ST hand-piece irradiation seem not induce an effective process, which did not lead to a matrix deposition while the FT profile showed a significant effect. Notwithstanding the limitation previously described, the literature supports our results by demonstrating the photobiomodulation positive effects on cell differentiation, which evidenced by increased the expression of Runx2 and ALP after irradiation with different wavelengths, such as: 632 nm (39), 659 nm (40), 830 nm (34), 904–910 nm (41), and 1064 nm (42) while the 780 nm inhibits them (43). However, in literature laser irradiation protocols and the late markers of the osteoblast differentiation remain controversial due to the poor evidence-based research documentations.

It is well known that the mature osteoblasts produce the organic components of the matrix constituted by Col 1a1, OC, osteopontin, osteonectin and bone sialoproteins (44). In Saracino et al. (41) work showed that the range of wavelength between 904 and 910 nm stimulated the OC. Moreover, Petri et al. (43) showed that the 780 nm laser irradiation had a positive effect on OC but the contrary on the Runx2 expression. This clearly demonstrates that there is no consistency in literature documentations on the effect of laser therapy on the Col 1a1 (12). However, our immunocytochemical data showed that the OC and the Col 1a1 were also up-regulated in cells treated 3 times a week for 2 weeks (total energy of 330 J) with both hand-pieces. However, further increase in these proteins was more consistent with the FT hand-piece irradiation. In addition, once a week for 2 weeks (total dose of 110 J) irradiation protocol with the FT hand-piece had a great effect on the Col 1a1 expression.

The signaling network that governs the osteoblasts maturation involves several intrinsic or extrinsic players. Nowadays, the Wnt signaling is considered as one of the most important local regulators of bone formation (45, 46). In osteoblast precursors, activation of the Wnt/βcatenin signaling increases the expression of osteogenic markers as well as the Wnt canonical pathway. This acts by the binding of the receptor, frizzled (Fz), and low-density lipoprotein receptor-related protein-5 or 6 (LRP-5/6) co-receptors. It's worth mentioning that a loss and a gain of the function mutations in the LRP5 would both lead to osteoporosis-pseudoglioma syndrome and high bone mass conditions (47, 48). In addition, TGFβ and Smads pathway play an important role in controlling the osteoblasts differentiation (49, 50). Our results have shown that the ST and FT hand-pieces were able to increase both the βcatenin and the Smads 2/3 without any effect on the expression of Smads 1/5/8, which was only noted when the cells were irradiated 3 times a week (total energy of 330J). Moreover, the expression of TGF-β was increased by the same total energy only with FT hand-piece irradiation. It has been demonstrated that Smads 3 enhances the ALP activity, the mineralization and the Col 1a1 in MC3T3-E1 pre-osteoblasts (51). Also, it was noted that TGFβ signaling promotes the osteoprogenitor proliferation, an early differentiation and commitment to the osteoblastic lineage in particular, the binding of TGFβ to the cognate receptors, which induces Smads phosphorylation (52). The later would be released from the receptor with consequent binding with other Smads. For instance, the Smads 2/3 association allows their translocation into the nucleus and the regulation of the gene transcription (28). Consequently, our laser therapy protocol of 3 times a week for 2 weeks at a total energy of 330J considered being effective in pre-osteoblast cell differentiation by activation of the Wnt signaling and the Smads 2/3 - βcatenin pathway with or without the TGFβ stimulation. While the BMP2/Smads 1/5/8 pathway, which is a potent inducer of Runx2, Osx and bone extracellular matrix proteins (28, 53) remained un-stimulated. Dang et al. (54) suggested that TGFβ/SMAD signaling pathway might play a role in PBM inducing collagen, when the skin was irradiated with 808 nm diode laser. However, the 808 and 980 nm seem to have different mechanisms of action at the cellular level (55). Notably, 808 nm has a primary target, the cytochrome c oxidase (CCO), which comprises unit IV in the mitochondrial respiratory chain (18, 55) while the 980 nm laser therapy can activate the transient receptor potential (*TRP*), multigene superfamily encodes integral membrane proteins, that acts on the ion channels (heat or light-gated ion channels) by heating up the microscopic regions of the water in the channel (15, 55). The identification of the primary target of 980 nm laser irradiation is not the topic of our work, however it is worth giving it consideration within our discussion. Some studies (56) showed that the TRP multigene superfamily comprises the Ca^+2^ channel and detected in a variety of non-neuronal tissues, including the chondrocytes and osteoclasts/osteoblasts. For instance, the TRP-valinoid (V) 4 is highly expressed in chondrocytes and it is important for the normal development of the bone growth plates (56, 57). In addition, other TRP channels can regulate the bone formation and mineralization (57). Moreover, many authors reported that many TRP channels have roles in cell proliferation, apoptosis, and differentiation in a variety of cell types (57). For instance, the TRPV1 deletion caused reduction in the expression of Runx2 and ALP in the bone marrow stromal cells and reduction in the calcium deposition *in vitro*, which would impair the fracture healing and inhibit the osteoclastogenesis and osteogenesis (58). If we consider that the 980 nm laser therapy in a range of energy comparable with our experimental setup, induces a fluctuation of stored Ca^2+^ and Ca^2+^ membrane fluxes in *Paramecium primaurelia* (21, 22), then the TRP genes will be found in the *Paramecium sp*., as their basic mechanism has evolved long before the appearance of multicellular animals. The PBM effect of the 420 nm and 540 nm encourages the osteoblastic-differentiation of human adipose-derived stem cells by the role of intracellular calcium TRP ion channels (55). The primary effect of our 980 nm laser therapy on water and TRP ion channels to launch a modulation of proliferation as well as a Wnt signaling may occur. This could be considered for future investigation.

Finally, our previous works [see reviews by Amaroli et al. (15) and Hamblin et al. (20)] suggested that the negative effect of higher fluences observed to date are not unequivocally due to high fluence *per se* but might be a consequence of the irradiation carried by ST profile of the handpiece. Inevitably, the results of the current study confirmed the great effects of the FT profile irradiation at a higher fluence on the pre-osteoblast differentiation in comparison to the ST profile.

## Conclusion

Our data, for the first time, prove that laser irradiation of 980 nm wavelength with FT beam profile delivery system in comparison to ST profile has a great photobiomodulatory efficacy on pre-osteoblastic cells differentiation, which would assist in accelerating bone regeneration, due to its homogeneous energy distribution at each point of its cross-section. Moreover, our results, for the first time, showed that the 980 nm laser therapy at effective and therapeutic parameters at ~0.9 W, ~0.9 W/cm^2^, 60 s, ~55 J/cm^2^ and a single energy ~55 J in CW improves the MC3T3-E1 pre-osteoblast viability and differentiation by inducing the Wnt signaling and the Smads 2/3—βcatenin pathway. Also, our data demonstrated that the dose-response and the magnitude of the 980 nm laser therapy effects vary with the total of energy administered. This is predictable but in a non-linear mode. Furthermore, the current study indicated and valued the intra-cellular pathways of the photon-cell interaction across the metabolic, proliferative and differentiation changes in the cells.

## Author Contributions

RH and GL performed the experiments. AA, DA, and MS designed the experiments, analyzed the data, and wrote the manuscript. SF wrote the manuscript. SB proposed the work and designed the experiments. FL and VC revised the paper.

### Conflict of Interest Statement

The authors declare that the research was conducted in the absence of any commercial or financial relationships that could be construed as a potential conflict of interest.
